# Mitochondrial Fuel Dependence on Glutamine Drives Chemo-Resistance in the Cancer Stem Cells of Hepatocellular Carcinoma

**DOI:** 10.3390/ijms22073315

**Published:** 2021-03-24

**Authors:** Alan Chun Kit Lee, Pui Man Lau, Yiu Wa Kwan, Siu Kai Kong

**Affiliations:** 1 School of Life Sciences, The Chinese University of Hong Kong, Shatin, N.T., Hong Kong, China; cklee@link.cuhk.edu.hk (A.C.K.L.); irenelau@cuhk.edu.hk (P.M.L.); 2 School of Biomedical Sciences, The Chinese University of Hong Kong, Shatin, N.T., Hong Kong, China; yiuwakwan@cuhk.edu.hk

**Keywords:** chemo-resistance, cancer stem cells, metabolic alteration, cancer cell metabolism, P-glycoprotein, mitochondria, hepatocellular carcinoma

## Abstract

Chemo-resistance hinders treatment of patients with hepatocellular carcinoma. Although there are many models that can be found in the literature, the root mechanism to explain chemo-resistance is still not fully understood. To gain a better understanding of this phenomenon, a chemo-resistant line, R-HepG2, was developed from a chemo-sensitive HepG2 line through an exposure of doxorubicin (DOX). The R-HepG2 exhibited a cancer stem cell (CSC) phenotype with an over-expression of P-glycoprotein (P-gp), conferring it a significant enhancement in drug efflux and survival. With these observations, we hypothesize that metabolic alteration in this drug-resistant CSC is the root cause of chemo-resistance. Our results show that, unlike other metabolic-reprogrammed CSCs that exhibit glycolytic phenotype described by the “Warburg effect”, the R-HepG2 was metabolically quiescent with glucose independence, high metabolic plasticity, and relied on glutamine metabolism via the mitochondria for its chemo-resistance Intriguingly, drug efflux by P-gp in R-HepG2 depended on the mitochondrial ATP fueled by glutamine instead of glycolytic ATP. Armed with these observations, we blocked the glutamine metabolism in the R-HepG2 and a significant reduction of DOX efflux was obtained. We exploited this metabolic vulnerability using a combination of DOX and metformin in a glutamine-free condition to target the R-HepG2, resulting in a significant DOX sensitization. In conclusion, our findings highlight the metabolic modulation of chemo-resistance in CSCs. We delineate the altered metabolism that drives chemo-resistance and offer a new approach to target this CSC through metabolic interventions.

## 1. Introduction

Chemotherapy is one of the first-line treatment strategies for many forms of cancer. The clinical outcomes of chemotherapy are generally satisfactory at the initial phase, but the efficiency of chemotherapeutics will inevitably be diminished due to the development of chemo-resistance [1]. Research has well-demonstrated the mechanisms of chemo-resistance, including but not limited to the dysregulation of pro-survival pathways and/or tumor suppressor genes, enhanced anti-apoptotic responses, over-expression of drug efflux transporters, alterations of mitochondria and strengthened genomic repair systems [2]. In addition, accumulating studies have identified a population of cancer stem cells (CSCs) as the major contributor of chemo-resistance, which raises the challenge of achieving complete tumor eradication and preventing cancer recurrence [3]. CSCs are a subgroup of cancer cells standing on the apex niche of the tumor cell population, possessing the capacity to self-renew, repopulate to heterogeneous lineages in tumor, and resist a broad range of chemotherapeutics or irradiation, conferring on them superior advantages over other cancer cells in survival even after a massive elimination of the tumor bulk by chemotherapeutic agents or radiation [4]. A widely applied approach to identify and characterize CSCs is based on the expression of multiple cell surface markers [5]. Despite that the origin of CSCs is still under debate, a consensus has converged that chemo-resistance can be effectively overcome by targeting the CSC population [6].

Altered metabolism and high metabolic plasticity are frequently observed in CSCs and, therefore, their metabolic phenotype is distinctive to the side population in tumor [7]. The altered metabolic phenotype of CSCs is diverse among different types of cancer, which can be either oxidative phosphorylation- (OXPHOS)-dependent [8] or highly glycolytic [9]. The chemo-resistance and stemness of CSCs are tightly controlled by the altered metabolism and metabolic plasticity [7,10]. Therefore, it is imperative to characterize the metabolic programs in CSCs. In general, switching of metabolic programs is co-mediated by glycolytic enzymes and mitochondria, in addition to a wide range of signaling pathways [11]. Dysregulation of mitochondrial functions [12], mitochondrial dynamics [13], and mitochondrial genome [14] are attributed to the altered metabolic phenotype in CSCs. As a result, mitochondria-specific pharmacological approaches have been used to target the CSCs’ mitochondria [5]. In fact, several models, including the highly malignant and recurrent glioblastoma and triple negative breast cancer CSCs, have been effectively sensitized to chemotherapy or radiotherapy by the mitochondria-specific or combinatory metabolic inhibition in in vitro and preclinical models [15,16]. Therefore, engagement of metabolic interventions as adjuvants to classical chemotherapeutics and radiotherapy pave a promising direction to overcoming chemo-resistance in cancers.

Hepatocellular carcinoma (HCC) ranks the fourth of cancer-related mortality globally. To date, doxorubicin (DOX) is still the mostly applied chemotherapeutic in the treatment of intermediate or advanced stage HCC. However, drug induced chemo-resistance in HCC is frequently developed during chemotherapy and is a huge hurdle in the cancer treatment [17]. Our research group has previously established a chemo-resistant line called R-HepG2 from the chemo-sensitive HepG2 through DOX incubation, and this chemo-resistant line had been used as a model to target chemo-resistance in HCC in other studies [18].

In this study, we report that the chemo-resistant R-HepG2 exhibited a CSC phenotype and displayed an altered metabolism characterized by a metabolically quiescent state and low glucose dependence compared to the chemo-sensitive HepG2. We hypothesize that the CSC R-HepG2 underwent a metabolic transition resulting in an altered metabolic state and shifted its energy reliance on supplies other than glucose to drive its chemo-resistance. We delineated the altered metabolism of R-HepG2 by means of metabolic profiling and employed inhibition of several major metabolic pathways to study the relation between the altered metabolism and chemo-resistance. We discovered that the chemo-resistance of R-HepG2 relied on the supply of mitochondrial ATP (Adenosine triphosphate) fueled by glutamine. Inhibition of mitochondria by metformin and deprivation of glutamine significantly sensitized the R-HepG2 to the DOX treatment.

## 2. Results

### 2.1. Enhanced Chemo-Resistance Is Due to the Drug Efflux by P-Glycoprotein in the R-HepG2

**Figure 1:**
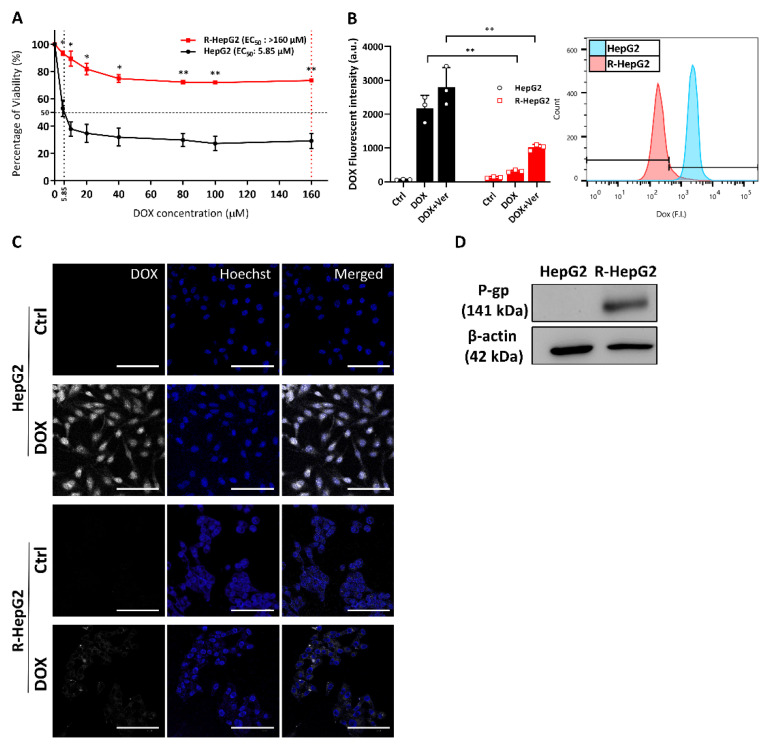
Enhanced chemo-resistance in the R-HepG2. (**A**) Cell viability under doxorubicin (DOX) treatment. Half maximal effective concentration (EC_50_) of DOX to cells is indicated. Experiments were performed with six technical replicates per group and results are shown as mean ± S.E.M. (*n* = 3). (**B**,**C**) DOX efflux of cells determined by fluorometric measurement of DOX (10 µM) by flow cytometry and confocal microscopy. P-gp blocker, Ver (100 µM), was added with DOX to confirm that the DOX efflux was mediated by P-gp. Results are presented as mean ± S.D. (*n* = 3). *t*-test was performed to compare HepG2 and R-HepG2 group. *: *p* ≤ 0.05; **: *p* ≤ 0.01. Scale bar: 100 µm. (**D**) An over-expression of P-gp (antibody dilution: 1:500) in the R-HepG2 was confirmed by immunoblotting.

The chemo-resistant line R-HepG2 was developed from the chemo-sensitive HepG2 as previously reported [18]. In brief, chemo-sensitive HepG2 cells were incubated with a stepwise increase of DOX concentration starting from 0.1 μM. The selection was done until the DOX concentration reached 100 μM. The survival population (i.e., R-HepG2) was maintained in 1.2 μM of DOX during cell passages throughout the entire period of study. R-HepG2 showed a significantly high tolerance to DOX treatment with an EC_50_ over 160 μM while the EC_50_ of HepG2 was 5.85 μM (Figure 1A). We found that the DOX efflux of R-HepG2 was significantly increased (i.e., reduction in cellular DOX) compared to the chemo-sensitive HepG2. The DOX efflux was reduced when R-HepG2 was co-treated with a P-glycoprotein (P-gp) blocker, verapamil (Ver, Figure 1B,C). Immunoblotting confirmed an over-expression of the ATP-binding cassette transporter (ABC transporter), P-gp which mediated DOX efflux in the R-HepG2, while its parental HepG2 cells did not express any P-gp (Figure 1D).

### 2.2. Chemo-Resistant R-HepG2 Is Enriched with CSC Markers

**Figure 2:**
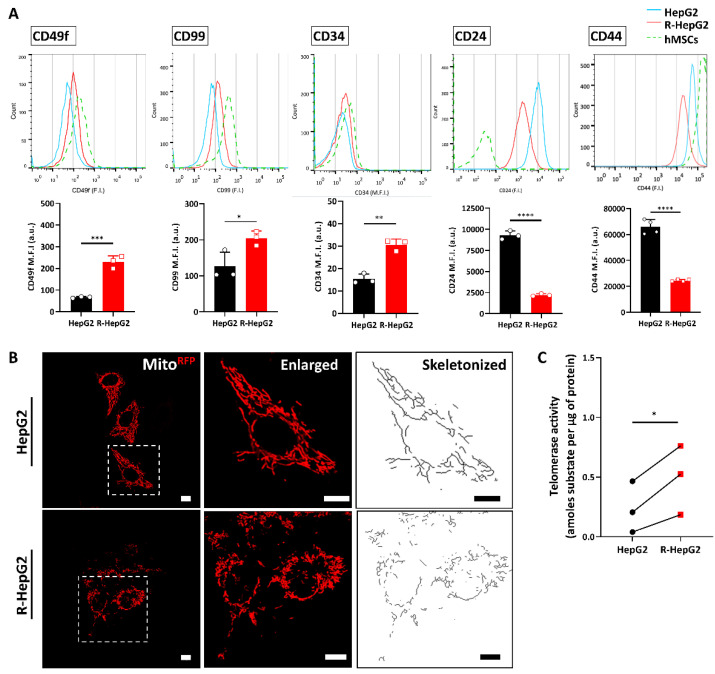
Characterization of cancer stem cell (CSC) phenotype in the R-HepG2. (**A**) CSC surface markers, CD49f, CD99, CD34, CD24 and CD44 were determined by cell surface immunofluorescent staining. Mean fluorescence intensity (M.F.I.) of the surface markers was quantified and shown in bar charts. Human mesenchymal stem cells (hMSCs) were used as a reference. Results are presented as mean ± S.D. (*n* = 3 or 4). *t*-test was performed to compare between HepG2 and R-HepG2 group. *: *p* ≤ 0.05; **: *p* ≤ 0.01; ***: *p* ≤ 0.001; ****: *p* ≤ 0.0001. (**B**) Representative confocal images showing the morphology of the red fluorescent protein- (RFP)-labelled mitochondria in HepG2 and R-HepG2 were chosen from three independent experiments. The mitochondrial network was skeletonized by ImageJ software for comparison. Scale bar: 10 µm. (**C**) Telomerase activity of HepG2 and R-HepG2 was determined by real-time PCR. Paired results from three biological samples are shown. *t*-test was performed to compare between HepG2 and R-HepG2 group. *: *p* ≤ 0.05.

Over-expression of P-gp is one of the distinctive markers of CSCs [2]; we hence questioned whether the R-HepG2 exhibited the CSC phenotype. Since CSCs cannot be identified by a single marker, we further verified the R-HepG2 with several well-established CSC surface markers by cell surface immunofluorescent staining, a common method for stem cell identification [5]. To compare the stem cell traits, a widely recognized stem cell, human mesenchymal stem cell (hMSC), was used as a reference. Results showed that the R-HepG2 exhibited a high level of CD49f, CD99, CD34 and a low level of CD24 and CD44 expression compared to its parental HepG2 (Figure 2A). We therefore characterized the phenotype of R-HepG2 as CD49f^+^, CD99^+^, CD34^hi^, CD24^low^ and CD44^low^. In addition to this, we generated lines of HepG2 and R-HepG2 constitutively expressing the mitochondria-targeted red fluorescent protein (mito^RFP^) to examine the mitochondria of these cells. We found that the R-HepG2 displayed small, discretely distributed, and fragmented mitochondria with a poorly developed mitochondrial network (Figure 2B), which is a typical characteristic of CSCs [11]. We have also studied the telomerase activity of HepG2 and R-HepG2, which is an indicator for the proliferative potential of CSCs [19]. The telomerase activity of the R-HepG2 was found to be higher than that of the HepG2 (Figure 2C). In addition to this, we observed that the chemo-resistance and P-gp expression of R-HepG2 were gradually ameliorated when DOX was removed from culture medium during passages (unpublished data), indicating the potential of R-HepG2 to differentiate into chemo-sensitive progenitor cells (i.e., heterogeneous differentiation) and thereby repopulating the tumor bulk during the breaks in chemotherapy. Together with the over-expression of P-gp, these data indicate that the chemo-resistant R-HepG2 exhibits the CSC phenotype.

### 2.3. R-HepG2 Has Low Glucose Dependence and Low Endogenous ATP Content

**Figure 3:**
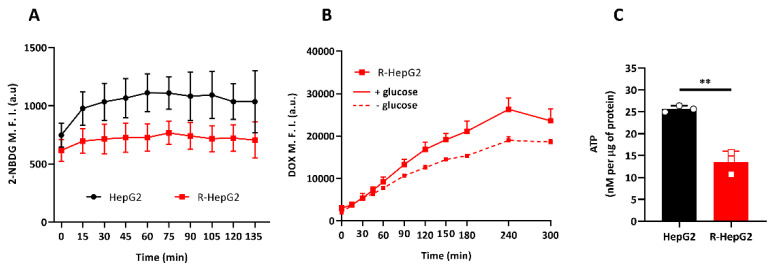
Low glucose dependence and low level of ATP (Adenosine triphosphate) in the R-HepG2. (**A**) Glucose uptake of cells over a 120 min course was determined by fluorometric measurement with a fluorescent glucose analogue, 2-NBDG (2-(*N*-(7-Nitrobenz-2-oxa-1,3-diazol-4-yl)Amino)-2-Deoxyglucose) by flow cytometry. Cells were incubated in a glucose-free RPMI 1640 medium for 30 min to depleted endogenous glucose before assay and experiments were performed in the presence of 5 μM of 2-NBDG. M.F.I. of the intracellular 2-NBDG at indicated time points was quantified and plotted against time. Results are expressed as mean ± S.D. (*n* = 3). (**B**) Drug efflux of the R-HepG2 determined with 10 µM of doxorubicin (DOX) in the absence or presence of glucose (11.1 mM, a formulated concentration of RPMI 1640 medium). Results from flow cytometry (M.F.I.) are expressed as mean ± S.D. (*n* = 3). (**C**) Absolute ATP content was determined by luciferase-based chemiluminescence. ATP content of lysate was calculated from the ATP standard with known concentration and normalized with the total protein concentration. Results are expressed as mean ± S.D. (*n* = 3). *t*-test was performed to compare between HepG2 and R-HepG2 group. **: *p* ≤ 0.01.

Knowing that the altered metabolism is a common feature in chemo-resistant CSCs [3,4,20], we decided to investigate the metabolic profile of R-HepG2 with reference to its parental HepG2. Base on the poorly developed mitochondrial network of R-HepG2, we expect that R-HepG2 relies heavily on glucose via glycolysis for energy supply. To test this hypothesis, we monitored the cellular glucose uptake at different time points by flow cytometry with a fluorescent glucose analogue, 2-(*N*-(7-Nitrobenz-2-oxa-1,3-diazol-4-yl)Amino)-2-Deoxyglucose (2-NBDG), over a 120 min course. To our surprise, we found that the glucose demand of R-HepG2 was significantly lower than that of HepG2 (Figure 3A). It is worthy of note that the endogenous glucose content at the beginning of the experiment (t = 0 min) was low in the R-HepG2. In contrast to HepG2, the uptake of glucose of R-HepG2 remained steadily low during the whole course of experiment. Interestingly, when R-HepG2 was treated with DOX in the absence of glucose, the intracellular DOX amount was lower than that with glucose, indicating that drug efflux was gradually enhanced and the energy for the drug efflux was not fueled directly by glucose (Figure 3B). We next measured the total endogenous ATP level by chemiluminescence, which reflected the metabolic status. Results in Figure 3C showed that R-HepG2 has a low ATP level, implicating a low metabolism. Taken together, these results indicate that the R-HepG2 had a low glucose demand and the drug efflux is independent of the availability of glucose, and should rely on metabolites other than glucose.

### 2.4. R-HepG2 Displays a Distinctive Metabolic Programme to Its Parental HepG2

Owing to its low metabolism reflected by the ATP content, we were curious to study the metabolic profile of R-HepG2. We determined the glycolysis rate and mitochondrial OXPHOS by measuring the extracellular acidification rate (ECAR) and the oxygen consumption rate (OCR) [21].

To determine the glycolysis rate, glucose-starved cells were firstly exposed to glucose to measure the basal glycolysis. Subsequently, mitochondrial OXPHOS was inhibited by oligomycin (Oligo), leading to a complete shift of metabolic program to glycolysis. The increase in ECAR represented the glycolysis reserve (i.e., a measurement of cells’ ability to increase glycolytic flux in response to high energetic demands for ATP production) for cells in response to the complete shutdown of OXPHOS. Finally, glycolysis was suppressed by a glucose analogue, 2-deoxy-D-glucose (2-DG), and the resulting change in ECAR represented the glycolytic capacity (i.e., a measurement of the maximum rate of conversion of glucose to pyruvate or lactate) of cells.

For the measurement of OCR, OXPHOS was sequentially inhibited by mitochondria toxicants including Oligo, carbonyl cyanide *p*-tri-fluoro-methoxy-phenylhydrazone (FCCP), and a mixture of rotenone and antimycin A (R/A). The electron transport chain (ETC) complex activities were inhibited and the resulted changes in OCR represented the OXPHOS rate. To ensure the assay accuracy, we included a rat heart myoblast cell, H9c2, as a positive control to validate the experimental condition.

**Figure 4:**
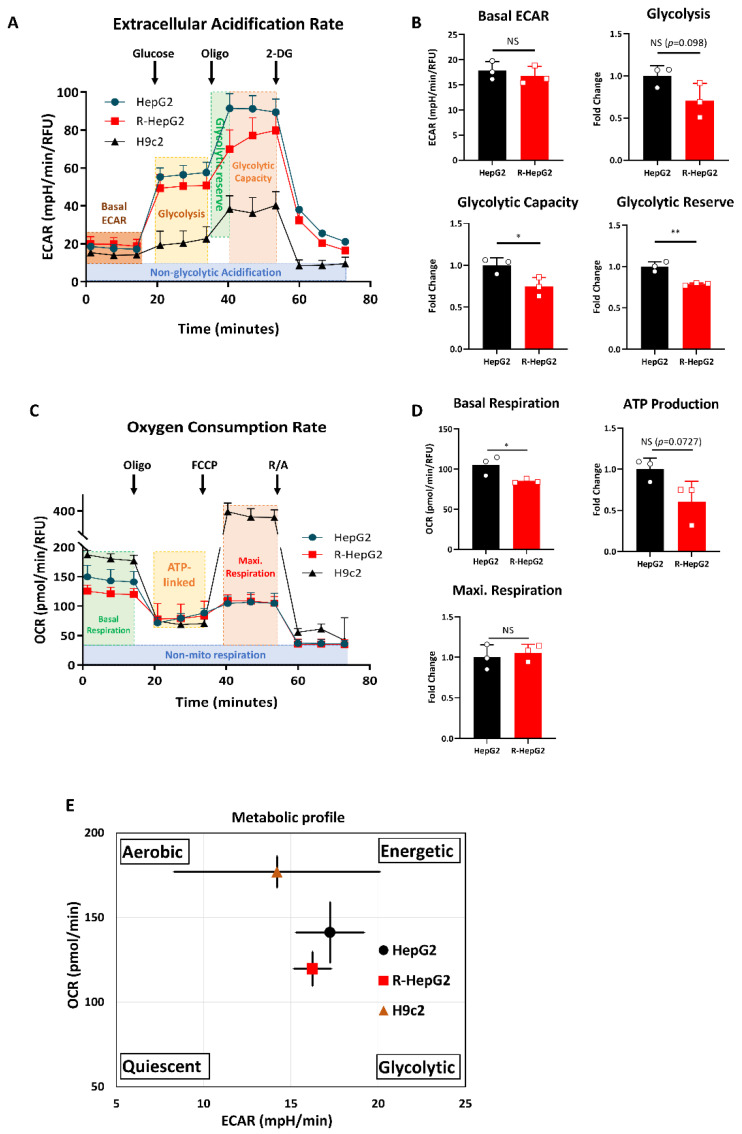
R-HepG2 displayed a metabolic quiescent state characterized by a low glycolysis and a low oxidative phosphorylation (OXPHOS)**.** (**A**) Glycolysis rate of cells was measured in terms of extracellular acidification rate (ECAR). Cells were sequentially treated with 10 mM of glucose, 1 μM of oligomycin (Oligo) and 50 mM of 2-deoxy-D-glucose (2-DG) to measure glycolytic responses. Parameters indicating their glycolytic ability were illustrated on the graph. (**B**,**D**) Parameters of glycolysis rate and OXPHOS rate were calculated from the graph and presented as bar charts. (**C**) OXPHOS rate of cells was measured in terms of oxygen consumption rate (OCR). Inhibitors of the electron transport chain (ETC) complexes, including 1 μM of Oligo, 1.5 μM of *p*-tri-fluoro-methoxy-phenylhydrazone (FCCP) and 1 μM of mixture of rotenone and antimycin A (R/A) were added to cells at indicated time points to measure their cellular responses. Parameters indicating their OXPHOS potential are illustrated on the graph. Rat heart myoblast, H9c2, was used as a positive control in both ECAR and OCR measurements. (**E**) Graphical summary of metabolic profiles of HepG2, R-HepG2 and H9c2, which were derived from the basal OCR and ECAR ratio. R-HepG2 showed a relatively steady quiescent state. Experiments were performed with four technical replicates per group and results are shown as mean ± S.D. (*n* = 3). *t*-test was performed to compare between HepG2 and R-HepG2 group. *: *p* ≤ 0.05; **: *p* ≤ 0.01; NS: non-significant.

As shown in Figure 4A,C, the R-HepG2 showed a low glycolysis and OXPHOS profile compared to that of HepG2. Consistent with its low glucose dependence, R-HepG2 showed a lower glycolysis rate, glycolytic capacity and glycolytic reserve, as well as basal respiration compared to its parental HepG2 (Figure 4B,D). These observations suggest a relatively quiescent state as reflected by a low OCR/ECAR ratio (Figure 4E). Moreover, the ATP level of R-HepG2 was found to be approximately half that of HepG2 as evidenced by both the chemiluminescent and OCR measurements (Figure 3C and Figure 4D). It is noteworthy that the mitochondria of both HepG2 and R-HepG2 were working on their bioenergetic maximum at basal state since their maximal rate of respiration could not increase further to meet the increased energy demand. The basal respiration of the R-HepG2 was found to be lower than the HepG2. However, the mitochondria of R-HepG2 with lower basal respiration were able to reach the same respiration maximum as parental HepG2 (Figure 4D), indicating that the R-HepG2 mitochondria were more flexible under metabolic stress. On the other hand, this result also implies that the mitochondria of HepG2 are less flexible to cope with a surge of ATP demand and may opt to rely on glycolysis for ATP production, which was manifested by its relatively high glycolytic phenotype. Collectively, these findings demonstrate that the R-HepG2 displays a metabolic quiescent state characterized by low activity on both glycolysis and OXPHOS (Figure 4E).

### 2.5. Altered Metabolic Phenotype Drives Chemo-Resistance by Regulating Drug Efflux in the R-HepG2

**Figure 5:**
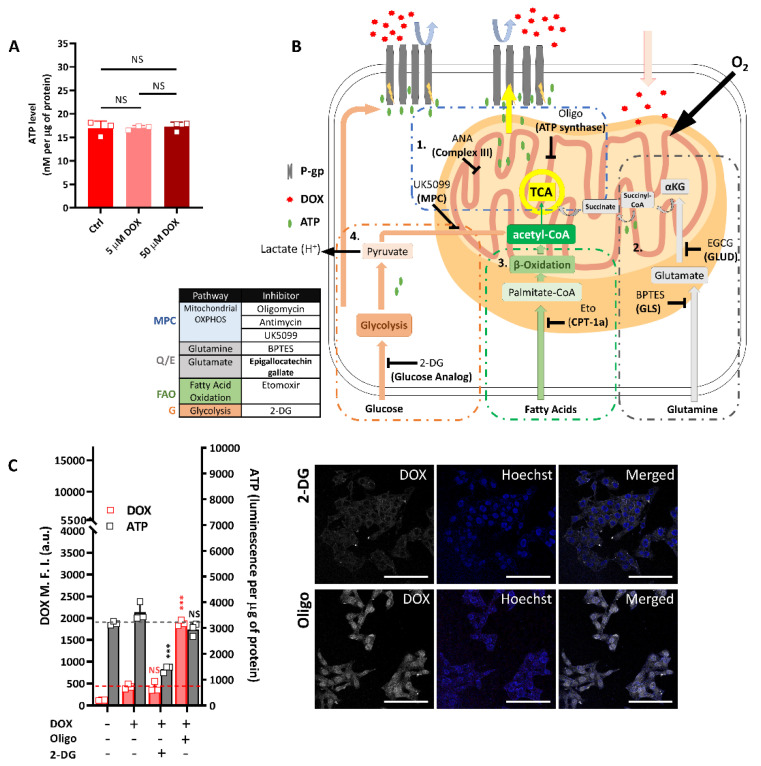

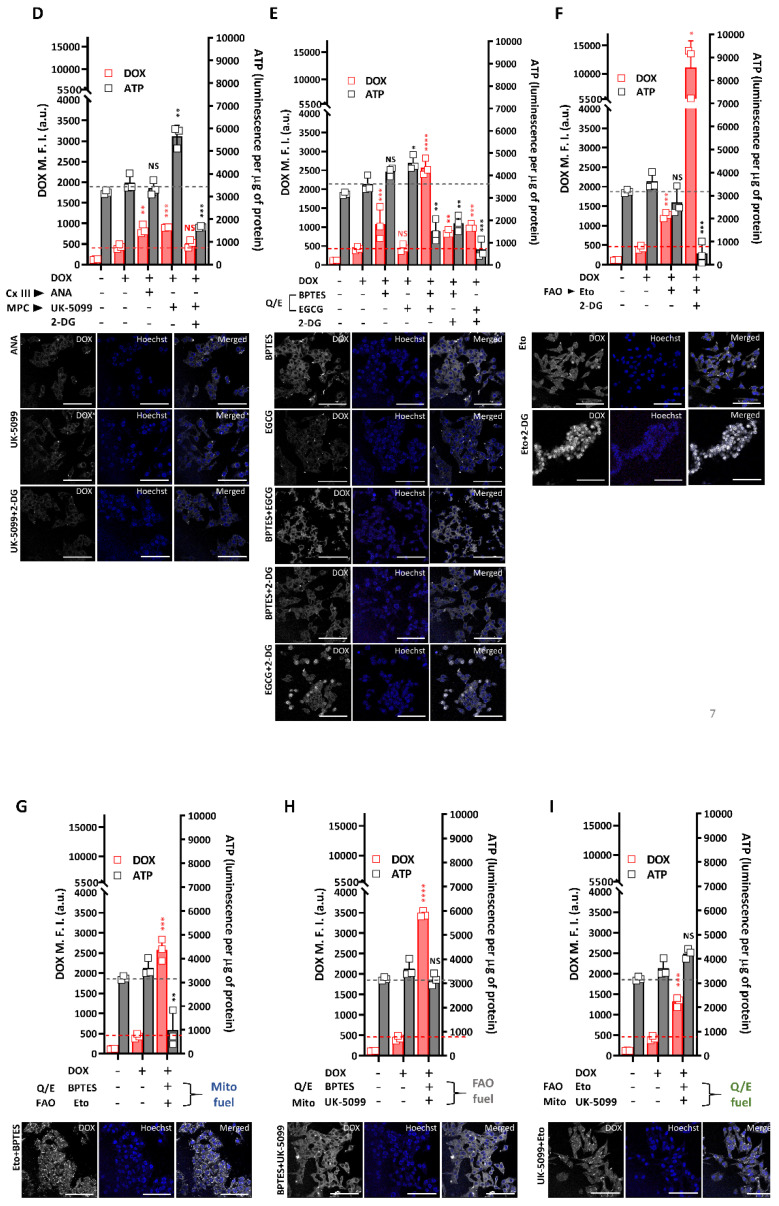
R-HepG2 relied on mitochondrial ATP for drug efflux. (**A**) ATP level of R-HepG2 remained unchanged after treatment with different doses of DOX. Results are shown as mean ± S.D. (*n* = 3). (**B**) Schema showing the major metabolic pathways and inhibitions. (**C**) Drug efflux and ATP content after co-treatment of DOX and metabolic inhibitor(s). The major metabolic pathways were inhibited by 50 mM of 2-DG (glycolysis), 5 μg/mL of oligomycin (Complex V), (**D**–**I**) 10 μg/mL of antimycin A (Complex III), 10 µM of UK-5099 (glucose-coupled MPC-pathway; mitochondrial pyruvate carrier), 10 µM of (Q/E pathway; glutaminase), 100 µM of epigallocatechin gallate (EGCG, Q/E-pathway; glutamate dehydrogenase), and 200 µM of etomoxir (Eto, FAO-pathway; carnitine palmitoyltransferase-1a). Red and black dotted lines denote the baseline value of the cellular DOX fluorescent signal and the ATP chemiluminescent signal of the DOX only group. Cells were treated with 10 µM DOX coupled with either in single or in dual combination of inhibitor(s) for 4 h at 37 °C. Corresponding media were used to avoid exogenous metabolic fuels. Drug efflux by cells after inhibition was imaged by a confocal microscope prior to flow cytometric measurement. Remaining cells were lysed for ATP measurement. Results are presented as mean ± S.D. (*n* = 3). *t*-test was performed to compare between the experimental and the DOX only control group. *: *p* ≤ 0.05; **: *p* ≤ 0.01; ***: *p* ≤ 0.001; ****: *p* ≤ 0.0001.; NS: non-significant. Scale bar: 100 µm.

Knowing the altered metabolic phenotype of the R-HepG2, we then asked whether this altered metabolism contributes to its chemo-resistance. Since the R-HepG2 over-expressed the multidrug resistance protein, P-gp, and based on prior knowledge of drug efflux, which is an energy consuming process relying on ATP hydrolysis [22], we tried to correlate drug efflux with ATP to establish a link between chemo-resistance and metabolic phenotype. Unexpectedly, the ATP level of R-HepG2 remained steady under the DOX treatment, regardless of the DOX input concentration (Figure 5A). Since the total ATP was produced from both glycolysis and the mitochondria, we hypothesized that this was a result of metabolic plasticity which allowed dynamic shifting between glycolysis-dependent and mitochondria-dependent ATP production within a low and constant ATP production profile [23]. To test this, we looked into the major metabolic pathways that fuel the metabolic requirement of cells (Figure 5B).

With the pharmacological interventions shown in Figure 5B, we inhibited the glycolysis and OXPHOS by 2-DG and Oligo, respectively. Glycolysis inhibition decreased the ATP content, whereas there was no significant change in OXPHOS inhibition when compared to that of the DOX only group (Figure 5C). As the OXPHOS was stopped upon inhibition of ATP synthase (complex V) by oligomycin, i.e., in the last step in ATP synthesis by mitochondrial OXHPOS, the comparable ATP level in the Oligo group to the DOX only control should be a result of compensation from glycolytic ATP and/or ATP produced from the glutamine, from the mitochondrial substrate level phosphorylation (mSLP). Conceivably, this compensation for the ATP loss from OXPHOS inhibition suggests a high metabolic plasticity of R-HepG2 that allowed it to survive better under metabolic challenges.

In Figure 5C, it is noteworthy to mention that the drug efflux represented by DOX fluorescence was significantly reduced (i.e., an increase in the cellular DOX M.F.I.) when OXPHOS complex V was inhibited by oligomycin, but the drug efflux remained unaffected under glycolysis inhibition. This finding indicated that drug efflux could not be restored even if there was an ATP compensation from glycolytic and/or mSLP. Although we did not include inhibition on the ATP synthesis from mSLP, the specific inhibition on the OXPHOS complex V of the mitochondria suggests that the drug efflux largely depended on the ATP produced from the mitochondrial OXPHOS, i.e., the mitochondrial ATP (Figure 5C).

Knowing that there are three metabolic pathways that fuel the mitochondria to produce ATP, namely the glucose-coupled mitochondrial OXPHOS pathway (MPC-pathway), the glutamine or glutamate-coupled OXPHOS pathway (Q/E pathway) and the fatty acid oxidation (FAO) -coupled OXPHOS pathway (FAO pathway), we extended our work by using inhibitors/blockers to target these pathways (Figure 5B).

The MPC-pathway fuels the mitochondria by importing pyruvate produced from glycolysis through the mitochondrial pyruvate carrier (MPC) to the mitochondrial matrix. The imported pyruvate is catalyzed to acetyl-CoA by pyruvate dehydrogenase and enters the TCA cycle in the mitochondria [24]. The Q/E-pathway fuels the mitochondria by the conversion of glutamine to glutamate by glutaminase (GLS) in the mitochondrial matrix or by a direct import of glutamate. The glutamate is then converted to α-ketoglutarate (αKG) by glutamate dehydrogenase (GLUD) and enters the TCA cycle. The oxidation of glutamine-derived αKG in the TCA cycle yields ATP by the catalysis of succinyl-CoA by succinate-CoA ligase (SUCL) in the absence or presence of oxygen through the mSLP. The resulting TCA intermediate succinate continues to be oxidized in the TCA cycle and yields reducing equivalents such as NADH and FADH_2_ for the OXPHOS of the ETC [23,25]. The FAO pathway fuels the mitochondria by importing long chain fatty acids through the carnitine palmitoyltransferase-1a (CPT-1a) to the mitochondrial matrix. The imported fatty acids undergo β-oxidation in the mitochondrial matrix and produce NADH and FADH_2_ reducing equivalents for the OXPHOS of the ETC and the TCA intermediate acetyl-CoA for the TCA cycle [26].

Consistent with our previous data, the ATP content remained unchanged or slightly increased when all pathways were inhibited by antimycin A (ANA; complex III), UK-5099 (MPC), BPTES (GLS), epigallocatechin gallate (EGCG; GLUD) and etomoxir (Eto; CPT-1a), whereas the ATP content decreased drastically when co-inhibited with 2-DG. These observations suggest again that the unchanged ATP level was due to an adaptive glycolytic compensation (Figure 5D–F).

When the DOX efflux was measured, notably all mitochondria-related inhibitions resulted in a reduced DOX efflux (i.e., an increase in the cellular DOX M.F.I.), regardless of the ATP compensation from glycolysis or mSLP, except the GLUD inhibition by EGCG. However, when GLS and GLUD were simultaneously inhibited, DOX efflux was significantly reduced (i.e., an increase in the cellular DOX M.F.I.), which implied that GLS was the key enzyme to regulate the Q/E pathway in the R-HepG2 (Figure 5E). When the glucose-coupled MPC-pathway (inhibited by UK-5099) was co-inhibited with glycolysis, a similar DOX efflux and ATP level to the glycolysis-only group (Figure 5C) was resulted (Figure 5D). This suggests the resultant ATP was produced from OXPHOS fueled by glutamine and/or fatty acids instead of glucose. This conclusion was also supported by the significantly reduced DOX efflux when both the Q/E- and FAO-pathway were inhibited (Figure 5E,F). To exclude the possibility that reduced DOX efflux was due to the diffusion of DOX across the leaky plasma membrane of a dead cell, we performed a trypan blue exclusion assay after metabolic inhibition to determine cell viability. Results showed that cells remained viable after 4 h of treatment (data not shown).

Based on these findings, we further delineated the mitochondrial fuel dependence of chemo-resistance. As glycolytic ATP did not contribute to DOX efflux, its effect on DOX efflux was negligible (Figure 5C). We simply used a dual inhibition on two of the three mitochondria fuels to restrict the mitochondria to a mono fuel format. First, co-inhibition of the Q/E- and FAO-pathway resulted in a mono fuel supply from the MPC-pathway. This dual inhibition resulted in a high cellular DOX fluorescence indicating a reduced DOX efflux (Figure 5G), thus ruling out the fuel dependence from the MPC-pathway. Likewise, the mono fuel supplied from the FAO-pathway resulted in a more significant reduction of DOX efflux (i.e., the highest cellular DOX content, Figure 5H), implying that fatty acids were not the mitochondrial fuel for DOX efflux. Lastly, reduction of DOX efflux was found to be least when there was a mono fuel supply from the Q/E-pathway, suggesting the primary mitochondrial fuel for DOX efflux was glutamine (Figure 5I). From these results, we concluded that the drug efflux in the R-HepG2 depended on the ATP produced from Q/E-mitochondrial OXPHOS. Taken together, the switch of mitochondrial fuel dependence from glucose to glutamine suggests a functional consequence of the altered metabolism in the R-HepG2.

### 2.6. R-HepG2 Is Senstized to DOX Treatment When the Mitochondrial OXPHOS Is Inhibited and Deprived of Glutamine Fuel

**Figure 6:**
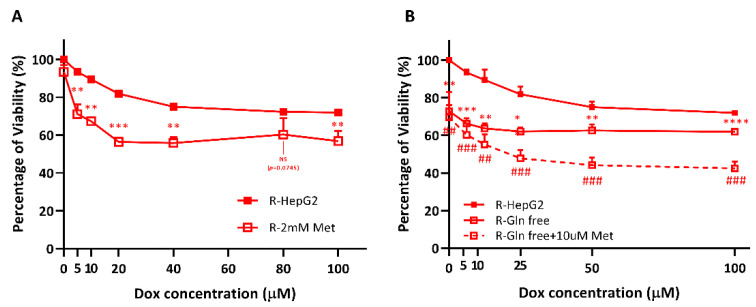
The cancer stem cell (CSC) R-HepG2 was effectively targeted by mitochondrial OXPHOS inhibition and glutamine deprivation. (**A**) Cells were co-treated with DOX and 2 mM of mitochondria-specific antagonist, metformin (Met) for 24 h, resulting in a significantly enhanced cytotoxicity. (**B**) The cytotoxicity was further enhanced in the glutamine-free RPMI 1640 medium (Gln-free) co-treated with metformin (10 µM) for 24 h. After treatments, cells were incubated with alamarBlue viability agent in RPMI 1640 medium for 1h. Experiments are performed with six technical replicates per group and results are shown as mean ± S.E.M. (*n* = 3). *t*-test was performed to compare between the control group and the experimental group. *: *p* ≤ 0.05; **/##: *p* ≤ 0.01; ***/###: *p* ≤ 0.001; ****: *p* ≤ 0.0001. NS: non-significant.

After confirming that the drug efflux of R-HepG2 depended largely on the mitochondrial ATP fueled by glutamine, we exploited this metabolic vulnerability of R-HepG2 using metformin, a primarily anti-hyperglycemic drug as well as a potent mitochondria-specific antagonist [27], to target the mitochondrial metabolism. Metformin specifically inhibits the mitochondrial ETC Complex I and causes a reduced NADH oxidation. This lowers the proton gradient across the mitochondrial membranes and hence reduces the proton-driven ATP synthesis by the mitochondria, resulting in a decreased mitochondrial ATP production [28]. As expected, results in Figure 6A showed that the R-HepG2 was significantly sensitized to DOX when co-treated with metformin. The cytotoxicity of DOX was further increased by approximately two-fold after a co-treatment with a low dose of metformin (10 µM) under a glutamine-staving condition (Figure 6B). It is of note that when the R-HepG2 was incubated in a glutamine-free condition, a pre-incubation death was observed, consolidating that glutamine, rather than glucose, was the essential metabolic fuel for the R-HepG2 (Figure 6B). Taken together, these results indicate that the chemo-resistance of R-HepG2 was driven by mitochondrial ATP fueled by glutamine, which was the functional consequence of its altered metabolism. Our results therefore demonstrate that this chemo-resistant CSC population can be effectively targeted by exploiting this metabolic vulnerability.

## 3. Discussion

Our understanding of cancer has been changed during the past decade. Conventional chemotherapy and radiotherapy targeted at tumor bulk eradication result in a differential killing and spare a small CSC population that contributes to chemo-resistance and recurrence [3]. The identification of CSCs and the revolutionary findings on the crosstalk between CSCs and cancer metabolism pave new routes to combat chemo-resistance. In this study, we developed a chemo-resistant line R-HepG2 from parental hepatocellular carcinoma HepG2 by an increasing dose of DOX incubation, a standard-of-care chemotherapeutic for HCC treatment, to mimic the clinical application. The R-HepG2 was able to survive in a high dose of DOX (EC_50_ > 160 µM) and was highly enriched with CSC markers and P-gp. The R-HepG2 is highly enriched with CD49f (integrin alpha 6), CD99 and CD34, which have been validated as robust CSC markers in previous studies [29,30,31]. Among them, CD49f directly regulates the stem cell determinants, OCT-4 and SOX-2 [32]. Additionally, R-HepG2 exhibits stem cell traits such as the poorly developed mitochondrial network that resembles the undifferentiated embryonic stem cells and mesenchymal stem cells [11], indicating its CSC phenotype. The fragmented mitochondria are generally recognized as bioenergetically compromised, which is deemed contradictory to the mitochondria-dependent chemo-resistant phenotype of R-HepG2. Nonetheless, we should consider the dynamic nature of mitochondria. The mitochondria are able to tether to a proximity of other organelles including nucleus and endoplasmic reticulum for communication or exchange of biomolecules [33,34]. The organelle tethering often results in a change of mitochondrial morphology and the mitochondria are not necessarily bioenergetically compromised. With regards to its dependence on mitochondrial ATP for drug efflux, it is possible that the fragmented mitochondria of R-HepG2 could be a result of mitochondrial tethering to the membrane-bound P-gp, though further investigation has to be conducted to test our hypothesis. Previous studies targeting chemo-resistance focused on the direct inhibition on drug efflux transporters such as the P-gp by various inhibitors [35], drug induction of mitochondrial intrinsic apoptotic pathway [36], mitochondria toxicants [37], modulation of signaling pathways [38] or inhibition on glycolysis [39]. Until recent years, the link between CSCs and chemo-resistance has been clarified [2]. For example, a number of researchers have reported a variety of chemo-resistant CSCs of different tumors displaying altered metabolic phenotypes characterized by either glycolytic- or OXPHOS-dependent metabolism. Moreover, a growing number of studies show that the CSC metabolism was rewired to metabolites other than glucose, such as glutamine [40].

It has been well-established that the over-expression of drug efflux transporters, such as P-gp, is a defense mechanism to resist chemotherapeutic insults in the CSCs [2,3]. It is widely accepted that this altered metabolism is essential to maintain the CSC stemness by regulating the expression of stemness-related genes, including OCT4 and MYC, or by enhancing the anti-oxidative ability conferred by the metabolic intermediates [7,10]. Nonetheless, how the altered metabolism in CSCs links to its chemo-resistance remains elusive. We herein demonstrated that the altered metabolic phenotype of the CSC R-HepG2 directly drove chemo-resistance by regulating drug efflux fueled by mitochondrial ATP through glutamine metabolism. In contrast to the observation from a previous study that inhibition of glycolytic ATP production could effectively target multidrug resistance in cancer cells [39], the chemo-resistance of R-HepG2 was insensitive to glycolysis inhibition. In fact, R-HepG2 displayed a high degree of glucose independence and transited to glutamine, as an alternative metabolite, to sustain energy requirement for its chemo-resistance. In addition to fueling the P-gp for drug efflux, this metabolic alteration results in two major advantages, one being the avoidance of intra-tumoral competition for glucose with the glucose-demanding and highly proliferating side population; the other being the control of redox homeostasis through generation of anti-oxidative derivatives, such as glutathione peroxidases and glutathione, as well as the key metabolic intermediate, αKG, for energy homeostasis [25]. Moreover, the low glycolysis and OXPHOS in the R-HepG2 implicates a dormant state that favors it in escaping from chemotherapeutics that are designed to target the highly proliferating and metabolically active tumor cells. By delineating the altered metabolic phenotype, we successfully sensitized the chemo-resistant CSC population through exploiting the metabolic vulnerability using a combined DOX treatment with the standard-of-care drug metformin and glutamine deprivation. In summary, our results show that simultaneous inhibition of the mitochondria and glutamine deprivation significantly sensitized R-HepG2 to DOX, suggesting an effective strategy to overcome chemo-resistance by exploiting this metabolic vulnerability of CSCs. We are aware of the limitations of the present study. For example, phenotypic plasticity is an emerging phenomenon that has been reported in some chemo-resistant cancer cell models [41]. Our investigations mainly focused on determination of the endpoint outcomes, which may largely overlook the dynamic phenotypic changes during treatment. Moreover, mitochondrial OXPHOS plays a vital role in mediating chemo-resistance [42] and CSC phenotype, the mitochondrial biology in the R-HepG2, remained largely uncharacterized in our study, which we should emphasize in future studies. Finally, despite the fact that R-HepG2 can be sensitized by exploiting the mitochondrial ATP through inhibition of glutamine influx, it is unknown whether the ATP produced from mSLP of glutamine would support its drug efflux. In particular, we did not include specific inhibitors to target the mSLP of glutamine in the present study. As a result, the ATP produced from mSLP of glutamine (also a form of mitochondrial ATP), in addition to ATP generated from OXPHOS of the ETC, might have simultaneously contributed to the drug efflux. The balance and the role of these two forms of mitochondrial ATP have to be determined in future study.

## 4. Materials and Methods

### 4.1. Reagents and Antibodies

Doxorubicin (DOX) was purchased from Enzo Life Sciences (Farmingdale, NY, USA). Metabolic inhibitors, MK-5099, etomoxir, and BPTES were purchased from MedChemExpress (Monmouth Junction, NJ, USA). EGCG, 2-DG, oligomycin, antimycin A, verapamil, protease inhibitor cocktails and anti-β-actin antibody (Cat-A2103) were purchased from Sigma (St. Louis, MO, USA). Bicinchoninic acid (BCA) protein assay kit, Hoechst 33342 nuclear stain solution, RIPA buffer, anti-P-gp antibody (Cat-MA1-26529) were purchased from Thermo Fisher Scientific (Waltham, MA, USA). Lipofectamine-3000 transfection reagent, 2-NBDG, HRP-conjugated goat anti-mouse IgG secondary antibody (Cat-31430) and alamarBlue cell viability reagent were purchased from Invitrogen (Carlsbad, CA, USA). Metformin, FITC-conjugated goat anti-mouse IgG secondary antibody (Cat-ab97039), PE-conjugated goat anti-mouse IgG secondary antibody (Cat-ab97041) and HRP-conjugated goat anti-rabbit IgG secondary antibody (Cat-ab97080) were purchased from Abcam (Cambridge, UK). Seahorse glycolysis-stress and mito-stress metabolic assay kits were purchased from Agilent Technologies (Santa Clara, CA, USA). Anti-CD24 (Cat-555426), anti-CD34 (Cat-555820), anti-CD44 (Cat-550392) anti-CD49f (Cat-555734), anti-CD99 (Cat-555687) antibodies were purchased from BD Biosciences (San Jose, CA, USA). FITC-conjugated goat anti-rat IgG secondary antibody (Cat-305002) was purchased from SouthernBiotech (Birmingham, AL, USA). An ATP quantification kit was purchased from Promega (Madison, WI, USA). A telomerase detection kit was purchased from Millipore (Burlington, MA, USA). All culture media including glucose-free and glutamine-free RPMI 1640 medium, Hanks’ balanced salt solution (HBSS), FBS, trypsin and antibiotics were purchased from Gibco (Carlsbad, CA, USA), unless otherwise specified.

### 4.2. Cell Culture and Transfection

HepG2 and H9c2 were purchased from American Type Culture Collection (ATCC). Human mesenchymal stem cell (hMSCs) was purchased from Gibco (Carlsbad, CA, USA). HepG2 and R-HepG2 were cultured in RPMI 1640 medium supplemented with 10% FBS and 1% penicillin/streptomycin antibiotics mixture (PS), except that R-HepG2 cells were supplemented with 1.2 μM of DOX in culture medium. H9c2 and hMSCs were cultured in DMEM and alpha-MEM supplemented with 10% FBS and 1% PS, respectively. hMSCs were assayed within four passages. To generate stable cell lines constitutively expressing the mitochondrial-RFP tag for mitochondria visualization, a construct pDsRed2-Mito (Clontech, CA, USA) carrying a human cytochrome C oxidase subunit VIII mitochondrial targeting sequence was transfected to cells using lipofectamine-3000 reagent according to manufacturer’s protocol. After 48 h of incubation, untransfected cells were eliminated by 1 mg/mL of Geneticin (InvivoGen, San Diego, CA, USA). To ensure clonal purity, transfected cells were sorted to a single clone by a cell sorter (FACSMelody, BD Biosciences, San Jose, CA, USA). Cells carrying the mitochondrial-RFP tag were expanded from a single clone. Fluorescence of the RFP-labelled mitochondria was verified by flow cytometry (FACSVerse, BD Biosciences, San Jose, CA, USA) and confocal microscopy (SP8, Leica, Wetzlar, Germany).

### 4.3. Cell Viability Assay

Cells (1 × 10^4^ per well) were seeded in a 96-well culture plate and incubated at 37 °C supplied with 5% CO_2_ overnight. Corresponding treatments were performed as indicated. After treatment, cells were washed with PBS and incubated with alamarBlue cell viability reagent in RPMI 1640 medium (1:9, *v*/*v*) for 1 h. Cells were subjected to fluorometric measurement by a multimode microplate reader (Tecan, Männedorf, Switzerland). Fluorescent readings of the treated group were normalized with the untreated group and presented as percentage of survival.

### 4.4. Drug Efflux, CSC Surface Marker Immunocytochemistry and Glucose Uptake by Flow Cytometry

For the drug efflux measurement, 0.5 × 10^6^ cells were seeded in a six-well culture plate and incubated overnight. Cells were treated with 5 μM of DOX only or co-treated with 100 μM of Ver for 4 h. After treatment, cells were loaded to the flow cytometer for DOX fluorescence measurement. For the CSC surface marker characterization, 1 × 10^6^ cells were incubated in the blocking buffer (1% BSA in PBS) for 30 min and were subsequently incubated with 0.5 μg of primary antibody for 30 min on ice, followed by a 30 min on ice incubation of 0.5 μg of fluorochrome-conjugated secondary antibody. After washing, cells were subjected to flow cytometric analysis. For the glucose uptake measurement, 0.5 × 10^6^ cells were incubated in glucose-free RPMI 1640 medium for 30 min to deplete endogenous glucose. After that, cells were incubated with 5 μM of 2-NBDG or with 10 μM of DOX in glucose-free RPMI 1640 medium. The fluorescent intensity of the 2-NBDG was measured with a 15 min time interval by a flow cytometer.

### 4.5. Telomerase Activity Assay

To access telomerase activity of HepG2 and R-HepG2, 1 × 10^6^ cells were lysed in 200 μL of CHAPS buffer and incubated on ice for 30 min. Lysates were centrifuged in 12,000 g for 20 min at 4 °C and the supernatant was collected. The protein concentration of the lysate was determined by BCA assay. Telomerase activity of cells was determined using the TRAPeze RT Telomerase Detection Kit. Reaction was set up consisting of 5 μL of TRAPeze RT Reaction Mix (5X), 17.6 μL of PCR grade water, 0.5 μL of 50X TITANIUM Taq DNA polymerase (Clontech, Mountain View, CA, USA) and 2 μL of cell extract (1 μg) or control template. Reactions were set up in triplicates and performed in a CFX96 real-time PCR system (Bio-Rad, Hercules, CA, USA) with the following cycling conditions: 30 min at 30 °C; 2 min at 95 °C followed by 45 cycles of 15 s at 94 °C, 60 s at 59 °C and 30 s at 45 °C. A standard reference for the telomerase activity was generated from serial dilutions (from 40 amoles to 0.04 amoles) of TSR8 positive control. A TSR8 standard curve was linearly plotted as log_10_ of the extended template quantity (amoles) versus the Ct values. The mean quantity (amoles) of the triplicates of the extended telomerase substrates produced from the samples’ telomerase was determined from the TSR8 standard curve. The obtained values were divided by the amount of protein (per μg) added in each reaction and expressed as amoles’ substrates per μg of protein.

### 4.6. Metabolic Profiling by Glycolysis-Stress and Mito-Stress Assays

Glycolysis-stress and mito-stress assays were performed to determine the metabolic and bioenergetic profiles of cells. Metabolic parameters were measured in terms of glycolysis and OXPHOS flux using a Seahorse XF Analyzer (Agilent Technologies, Santa Clara, CA, USA). In brief, 4 × 10^4^ cells were seeded in a 96-well microplate and incubated overnight. For glycolysis flux analysis, cells were pre-incubated in assay medium provided by the manufacturer for 30 min for endogenous glucose depletion. Cells were sequentially treated with 10 mM of glucose, 1 μM of oligomycin and 50 mM of 2-DG. Cellular responses were measured by the change in extracellular acidification rate (ECAR; mpH/min). For OXPHOS analysis, cells were sequentially treated with 1 μM of oligomycin, 1.5 μM of FCCP and 1 μM of mixture of antimycin A and rotenone. Cellular responses were measured by the change in oxygen consumption rate (OCR; pmol/min). After assay, cells were immediately fixed and permeabilized with ice-cold methanol:acetone mixture (1:1, *v*/*v*). Fixed cells were stained with 3.75 µg/mL of Hoechst 33343 nuclear stain. Nuclear fluorescence of stained cells was measured by microplate reader and was used to normalize the ECAR and OCR values.

### 4.7. Endogenous ATP Measurement

Endogenous ATP measurement was performed by a luciferase-based chemiluminescence assay kit. In brief, cells were lysed in 150 μL of lysis buffer and the lysates were equilibrated at room temperature for 10 min to allow the enzymatic reaction to proceed. Subsequently, 20 μL of lysate was added in triplicates and loaded to the microplate reader for chemiluminescence measurement. Absolute ATP quantity was calculated from a standard with known ATP concentration. The protein concentration of the lysate was determined by BCA assay and used to normalize the chemiluminescent signal.

### 4.8. Mitochondrial Fuel Dependence Assay

To evaluate the mitochondrial fuel dependence of chemo-resistance in the R-HepG2, the major metabolic pathways were inhibited by 50 mM of 2-DG, 5 μg/mL of Oligo, 10 μg/mL of ANA, 10 µM of UK-5099, 10 µM of BPTES, 100 µM of EGCG, and 200 µM of Eto. In brief, 3 × 10^5^ cells were treated with 10 µM of DOX coupled with either in single or in dual combination of inhibitor(s) for 4 h at 37 °C. The 2-DG group was incubated in glucose-free RPMI 1640 medium for 30 min to deplete endogenous glucose prior to assay. To avoid exogenous metabolic fuel, the treatment was performed in glucose-free RPMI 1640 medium. For the BPTES group, the treatment was performed in 1% BSA/HBSS for the same purpose. Drug efflux by cells after inhibition was imaged by a confocal microscope prior to flow cytometric measurement. The remaining cells were lysed for ATP measurement as described above.

### 4.9. Mitochondrial Morphology

Mitochondrial-RFP tagged cells (1 × 10^5^ cells per dish) were seeded in a confocal dish and incubated overnight. The mitochondria of live cells were imaged using a confocal microscope. The mitochondria skeletonized morphology was processed by Image J software (NCBI, Bethesda, MD, USA).

### 4.10. SDS-PAGE and Western Blotting

Total proteins were extracted from cell pellets by RIPA buffer (supplemented with 1% PMSF and 1% protease inhibitor cocktails, *v*/*v*). Protein concentration of lysates was determined by BCA assay. Lysates were denatured by 6X SDS solution (5% β-mercapto-ethanol, 0.02% bromophenol blue, 250 mM Tris-Cl (pH 6.8), 30% glycerol and 10% SDS, *v*/*v*) with 10 min of 95 °C heating. After that, 25 µg of samples were separated by SDS-PAGE and transferred to a polyvinylidene fluoride membrane for immunoblotting. The transferred membrane was blocked with a blocking buffer (3% BSA in PBS with 0.1% Tween-20, PBS-T) at room temperature for 1 h. After washes of PBS-T, corresponding primary antibody diluted in blocking buffer was added to the membrane and incubated at 4 °C overnight. On the next day, the membrane was probed with the HRP-conjugated secondary antibody at room temperature for 1 h. Protein bands were visualized by adding HRP substrate (Millipore, Burlington, MA, USA) to the membrane and the chemiluminescent signal was captured by a ChemiDoc Imaging System (Bio-Rad, Hercules, CA, USA). The β-actin was used as the internal loading control.

### 4.11. Statistical Analysis

Results are presented as mean ± S.D or mean ± S.E.M. Student’s *t*-test was performed to compare statistical significance between the experimental group and the control group. *p* values equal to or less than 0.05 (i.e., *p* ≤ 0.05) were considered as statistically significant. Statistical analyses were performed using the GraphPad Prism software (version 8; San Diego, CA, USA).

## Data Availability

Data not included within the manuscript are available from the corresponding author upon request.

## Abbreviations

αKG	α-ketoglutarate
2-DG	2-deoxy-D-glucose
2-NBDG	2-(*N*-(7-Nitrobenz-2-oxa-1,3-diazol-4-yl)Amino)-2-Deoxyglucose
ATP	adenosine triphosphate
BPTES	*bis*-2-(5-phenylacetamido-1,2,4-thiadiazol-2-yl)ethyl sulfide
DOX	doxorubicin
ECAR	Extracellular acidification rate
EGCG	Epigallocatechin gallate
Eto	Etomoxir
FCCP	carbonyl cyanide *p*-trifluoro-methoxy-phenyl-hydrazone
mSLP	mitochondrial substrate level phosphorylation
OCR	Oxygen consumption rate
P-gp	P-glycoprotein
UK-5099	2-Cyano-3-(1-phenyl-1H-indol-3-yl)-2-propenoic acid
Ver	Verapamil
OXPHOS	Oxidative phosphorylation

## References

[1] Alfarouk K.O., Stock C.M., Taylor S., Walsh M., Muddathir A.K., Verduzco D., Bashir A.H.H., Mohammed O.Y., Elhassan G.O., Harguindey S. (2015). Resistance to cancer chemotherapy: Failure in drug response from ADME to P-gp. Cancer Cell Int..

[2] Abdullah L.N., Chow E.K. (2013). Mechanisms of chemoresistance in cancer stem cells. Clin. Transl. Med..

[3] Cojoc M., Mäbert K., Muders M.H., Dubrovska A. (2015). A role for cancer stem cells in therapy resistance: Cellular and molecular mechanisms. Semin. Cancer Biol..

[4] Koren E., Fuchs Y. (2016). The bad seed: Cancer stem cells in tumor development and resistance. Drug Resist. Updat..

[5] De Francesco E.M., Sotgia F., Lisanti M.P. (2018). Cancer stem cells (CSCs): Metabolic strategies for their identification and eradication. Biochem. J..

[6] Nguyen L.V., Vanner R., Dirks P., Eaves C.J. (2012). Cancer stem cells: An evolving concept. Nat. Rev. Cancer.

[7] Sancho P., Barneda D., Heeschen C. (2016). Hallmarks of cancer stem cell metabolism. Br. J. Cancer.

[8] Sancho P., Burgos-Ramos E., Tavera A., Bou Kheir T., Jagust P., Schoenhals M., Barneda D., Sellers K., Campos-Olivas R., Graña O. (2015). MYC/PGC-1α balance determines the metabolic phenotype and plasticity of pancreatic cancer stem cells. Cell Metab..

[9] Shen Y.A., Wang C.Y., Hsieh Y.T., Chen Y.J., Wei Y.H. (2015). Metabolic reprogramming orchestrates cancer stem cell properties in nasopharyngeal carcinoma. Cell Cycle.

[10] Peiris-Pagès M., Martinez-Outschoorn U.E., Pestell R.G., Sotgia F., Lisanti M.P. (2016). Cancer stem cell metabolism. Breast Cancer Res..

[11] Peixoto J., Lima J. (2018). Metabolic traits of cancer stem cells. Dis. Model. Mech..

[12] Kim E.J., Jin X., Kim O.R., Ham S.W., Park S.H., Kim H. (2018). Glioma stem cells and their non-stem differentiated glioma cells exhibit differences in mitochondrial structure and function. Oncol. Rep..

[13] Chen H., Chan D.C. (2017). Mitochondrial dynamics in regulating the unique phenotypes of cancer and stem cells. Cell Metab..

[14] Guha M., Srinivasan S., Ruthel G., Kashina A.K., Carstens R.P., Mendoza A., Khanna C., van Winkle T., Avadhani N.G. (2014). Mitochondrial retrograde signaling induces epithelial-mesenchymal transition and generates breast cancer stem cells. Oncogene.

[15] Lee K.M., Giltnane J.M., Balko J.M., Schwarz L.J., Guerrero-Zotano A.L., Hutchinson K.E., Nixon M.J., Estrada M.V., Sánchez V., Sanders M.E. (2017). MYC and MCL1 cooperatively promote chemotherapy-resistant breast cancer stem cells via regulation of mitochondrial oxidative phosphorylation. Cell Metab..

[16] Oizel K., Chauvin C., Oliver L., Gratas C., Geraldo F., Jarry U., Scotet E., Rabe M., Alves-Guerra M.C., Teusan R. (2017). Efficient mitochondrial glutamine targeting prevails over glioblastoma metabolic plasticity. Clin. Cancer Res..

[17] Yang J.D., Hainaut P., Gores G.J., Amadou A., Plymoth A., Roberts L.R. (2019). A global view of hepatocellular carcinoma: Trends, risk, prevention and management. Nat. Rev. Gastroenterol. Hepatol..

[18] Cheung J.Y., Ong R.C., Suen Y.K., Ooi V., Wong H.N., Mak T.C., Fung K.P., Yu B., Kong S.K. (2005). Polyphyllin D is a potent apoptosis inducer in drug-resistant HepG2 cells. Cancer Lett..

[19] Ju Z., Rudolph K.L. (2006). Telomeres and telomerase in cancer stem cells. Eur. J. Cancer.

[20] Bosc C., Selak M.A., Sarry J.E. (2017). Resistance is futile: Targeting mitochondrial energetics and metabolism to overcome drug resistance in cancer treatment. Cell Metab..

[21] Van der Windt G.J.W., Chang C.H., Pearce E.L. (2016). Measuring bioenergetics in T cells using a seahorse extracellular flux analyzer. Curr. Protoc. Immunol..

[22] Wilkens S. (2015). Structure and mechanism of ABC transporters. F1000Prime Rep..

[23] Seyfried T.N., Arismendi-Morillo G., Mukherjee P., Chinopoulos C. (2020). On the origin of ATP synthesis in cancer. iScience.

[24] Herzig S., Raemy E., Montessuit S., Veuthey J.L., Zamboni N., Westermann B., Kunji E.R., Martinou J.C. (2012). Identification and functional expression of the mitochondrial pyruvate carrier. Science.

[25] Yang L., Venneti S., Nagrath D. (2017). Glutaminolysis: A hallmark of cancer metabolism. Annu. Rev. Biomed. Eng..

[26] Carracedo A., Cantley L.C., Pandolfi P.P. (2013). Cancer metabolism: Fatty acid oxidation in the limelight. Nat. Rev. Cancer.

[27] Andrzejewski S., Gravel S.P., Pollak M., St-Pierre J. (2014). Metformin directly acts on mitochondria to alter cellular bioenergetics. Cancer Metab..

[28] Vial G., Detaille D., Guigas B. (2019). Role of mitochondria in the mechanism(s) of action of metformin. Front. Endocrinol. Lausanne.

[29] Lathia J.D., Gallagher J., Heddleston J.M., Wang J., Eyler C.E., Macswords J., Wu Q., Vasanji A., McLendon R.E., Hjelmeland A.B. (2010). Integrin alpha 6 regulates glioblastoma stem cells. Cell Stem Cell.

[30] Chung S.S., Eng W.S., Hu W., Khalaj M., Garrett-Bakelman F.E., Tavakkoli M., Levine R.L., Carroll M., Klimek V.M., Melnick A.M. (2017). CD99 is a therapeutic target on disease stem cells in myeloid malignancies. Sci. Transl. Med..

[31] Saito Y., Kitamura H., Hijikata A., Tomizawa-Murasawa M., Tanaka S., Takagi S., Uchida N., Suzuki N., Sone A., Najima Y. (2010). Identification of therapeutic targets for quiescent, chemotherapy-resistant human leukemia stem cells. Sci. Transl. Med..

[32] Yu K.R., Yang S.R., Jung J.W., Kim H., Ko K., Han D.W., Park S.B., Choi S.W., Kang S.K., Schöler H. (2012). CD49f enhances multipotency and maintains stemness through the direct regulation of OCT4 and SOX2. Stem Cells.

[33] Desai R., East D.A., Hardy L., Faccendam D., Rigon M., Crosby J., Alvarez M.S., Singh A., Mainenti M., Hussey L.K. (2020). Mitochondria form contact sites with the nucleus to couple prosurvival retrograde response. Sci. Adv..

[34] Xia M., Zhang Y., Jin K., Lu Z., Zeng Z., Xiong W. (2019). Communication between mitochondria and other organelles: A brand-new perspective on mitochondria in cancer. Cell Biosci..

[35] Amin M.L. (2013). P-glycoprotein inhibition for optimal drug delivery. Drug Target Insights.

[36] Ong R.C., Lei J., Lee R.K., Cheung J.Y., Fung K.P., Lin C., Ho H.P., Yu B., Li M., Kong S.K. (2008). Polyphyllin D induces mitochondrial fragmentation and acts directly on the mitochondria to induce apoptosis in drug-resistant HepG2 cells. Cancer Lett..

[37] Wang H., Zhang F., Wen H., Shi W., Huang Q., Huang Y., Xie J., Li P., Chen J., Qin L. (2020). Tumor-and mitochondria-targeted nanoparticles eradicate drug resistant lung cancer through mitochondrial pathway of apoptosis. J. Nanobiotechnol..

[38] Yang R., Huang B., Zhu Y., Li Y., Liu F., Shi J. (2018). Cell type-dependent bimodal p53 activation engenders a dynamic mechanism of chemoresistance. Sci. Adv..

[39] Xu R.H., Pelicano H., Zhou Y., Carew J.S., Feng L., Bhalla K.N., Keating M.J., Huang P. (2005). Inhibition of glycolysis in cancer cells: A novel strategy to overcome drug resistance associated with mitochondrial respiratory defect and hypoxia. Cancer Res..

[40] Chen Q., Kirk K., Shurubor Y.I., Zhao D., Arreguin A.J., Shahi I., Valsecchi F., Primiano G., Calder E.L., Carelli V. (2018). Rewiring of glutamine metabolism is a bioenergetic adaptation of human cells with mitochondrial DNA mutations. Cell Metab..

[41] Goldman A., Khiste S., Freinkman E., Dhawan A., Majumder B., Mondal J., Pinkerton A.B., Eton E., Medhi R., Chandrasekar V. (2019). Targeting tumor phenotypic plasticity and metabolic remodeling in adaptive cross-drug tolerance. Sci. Signal.

[42] Ghosh P., Vidal C., Dey S., Zhang L. (2020). Mitochondria targeting as an effective strategy for cancer therapy. Int. J. Mol. Sci..

